# Chemical structure, properties and potential applications of surfactin, as well as advanced strategies for improving its microbial production

**DOI:** 10.3934/microbiol.2023012

**Published:** 2023-03-14

**Authors:** Cheng Zhen, Xian-Feng Ge, Yi-Ting Lu, Wen-Zheng Liu

**Affiliations:** School of Food and Pharmaceutical Engineering, Nanjing Normal University, Nanjing 210023, China

**Keywords:** surfactin, biological property, biosynthesis, genetic engineering, synthetic biology

## Abstract

Surfactin, a cyclic lipopeptide produced by microbes belonging to the genus *Bacillus*, is one of the most effective biosurfactants available in many industrial fields. However, its low production and high cost have intensively constrained its commercial applications. In this review, we first summarize the molecular structure, biological properties, beneficial roles and potential applications of surfactin in the fields of medical care and food safety, highlighting the great medical and commercial values of making its industrial production into reality. Further, genetic regulation for surfactin biosynthesis and advanced strategies for enhancing its microbial production, including optimizing fermentation conditions, rational genetic engineering and synthetic biology combined with metabolic engineering approaches, are elucidated. Finally, prospects for improving surfactin biosynthesis are discussed, and the establishment of suitable chassis hosts for exogenous production of surfactin might serve as an important strategy in future research.

## 1. Introduction

Pathogenic microorganisms, especially pathogenic bacteria, are microbial species that cause a variety of diseases in animals, plants and humans [1], resulting in huge economic losses worldwide every year [2]. Antibiotics serve as one of the main strategies for treating pathogenic bacterial infections, but this frequently allows the adaptive evolution of microbial species to have antibiotic resistance. In addition, many studies have demonstrated that most antibiotics could generate a range of side effects that can be life-threatening [3]. Therefore, it is essential to discover and develop new antimicrobial agents that are not resistant to therapeutic effects and have fewer side effects. Microorganisms have attracted much attention for their potential to produce multiple biologically active metabolites, among which biosurfactants are key targets for research focusing on developing novel antimicrobial agents due to their profound antibacterial and biological activities [4].

Surfactin is a lipopeptide-based bioactive substance produced by microbial species belonging to the genus *Bacillus*. As one of the most effective biosurfactants available, the numerous physiological and biochemical activities of surfactin have received considerable attention [5],[6]. According to reports, surfactin has pharmacological effects like antibacterial and antifungal properties [7] and anti-mycoplasma [8], antiviral [9], anti-inflammatory [10] and thrombolytic [9] activities. Surfactin displays an amphiphilic surface activity by the presence of hydrophobic and hydrophilic parts in its molecules, which allow them to aggregate at the interface of two immiscible liquids, thus reducing the interfacial tension of the liquid or different material phases [11]. Due to the good antimicrobial effect and safety in food applications, surfactin could serve as a candidate for inhibiting bacterial growth and maintaining the sensory status of food products during storage. This important property allows surfactin to play a potential role in food preservation [12]. In addition, some studies have pointed out implications of surfactin for improving composition and structure of intestinal microorganisms, indicating its potential application in ameliorating intestinal microbiota dysfunction [13].

Although surfactin has potential applications in many areas, it is currently not competitive with chemically synthetic surfactants due to the high cost and low yield of its production. Fermentation conditions have been intensively optimized, in order to improve the yield of surfactin. In addition, the use of genetic engineering means that improvement of surfactin production is gaining more and more attention. Moreover, the development of synthetic biology also holds great promises for further improvement of surfactin production [14].

In this paper, the structure, physiological and biochemical characteristics and antibacterial mechanisms of surfactin and its main applications are reviewed, and the advanced strategies to improve its microbial production are also presented in detail.

## 2. Structure and physicochemical properties of surfactin

The discovery history of surfactin could date back to 1968, when this biologically active secondary metabolite was first identified in cultures of *Bacillus subtilis* strains [15]. The structure of surfactin has a cyclic peptide chain with 7 amino acids and a 13–16 carbon atom hydroxy fatty acid chain, which together create a cyclic lactone ring structure. Positions 2, 3, 4, 6 and 7 are occupied by hydrophobic amino acid residues, whereas positions 1 and 5, by glutamyl and aspartyl residues, respectively, add two negative charges to the molecule. In cells, various surfactin isomers often coexist as a combination of various peptide variations with various aliphatic chain lengths [16].

Surfactin has considerable surface activity because of its amphiphilic nature, which also allows it to reduce surface/interfacial tension and self-assemble in the nanostructure. As a result, it shows physicochemical properties such as foaming [17], emulsification [18], solid surface drying prevention and chelating ability [19],[20]. Recently, it has been suggested that surfactin can also be effective in demulsifying waste crude oil [21]. Its emulsification properties also give it potential for applications in the food and cosmetic sectors, as well as in the pharmaceutical sector [22],[23].

Surfactin is considered an extremely effective surfactant molecule. At a concentration of 20 µM, it reduces the surface tension of water from 72 mN/m to 27 mN/m, which is approximately 2 log lower than those caused by most other detergents [24],[25]. In terms of aggregation activity, the critical micelle concentration (CMC) value of surfactin decreases continuously as its fatty acid chains become longer [26]. In general, the CMC of biosurfactants is lower than that of chemically synthetic surfactants. The low CMC of biosurfactants, especially surfactin, iturin A and fengycin, makes them very interesting for use in various fields because they are required in smaller quantities compared to petrochemical-derived surfactants [27]. In general, even microbial derived surfactin with low purity has a lower CMC than its synthetic counterpart, which is highly favorable for its application in bioremediation or petroleum recovery.

## 3. Surfactin-membrane interaction

The antibacterial activity of surfactin is closely related to its biofilms. Surfactin is an amphiphilic molecule that destabilizes membranes and disrupts their integrity [28]. The mechanisms of action are as follows: insertion into lipid bilayers, chelation of monovalent and divalent cations or modification of membrane permeability by formation of channels [29].

The arrangement of the hydrocarbon chains and the membrane's thickness are both impacted by surfactin's penetration of the membrane through hydrophobic interactions. The surfactin peptide cycle then exhibits structural changes following this initial collision, which contributes to the interaction process [30]. *In vitro*, surfactin binding to the membrane causes dehydration of the phospholipid polar head group and severely affects the stability of the bilayer, leading to disturbances in membrane barrier properties. This provides a good explanation for the antimicrobial effect of lipopeptides [31].

Both monovalent and divalent cations can be propelled by surfactin past organic barriers, with divalent cations doing so more effectively [32]. At the air/water interface, the two acidic residues Glu-1 and Asp-5 are partially neutralized by Na^+^ and K^+^, whereas complete neutralization is induced by Ca^2+^. The cation chelation could result in the inhibition of cyclic phosphodiesterase activity. Furthermore, by neutralizing the surfactin and lipid charges and maintaining the 1:1 surfactin-calcium complex, Ca^2+^ could enable the surfactin to penetrate the membrane more deeply with the aid of Glu-1 and Asp-5. This effect on the conformation of surfactin promotes deeper insertion of surfactin into the cell membrane and its antimicrobial effect [6].

## 4. Potential applications and beneficial roles of surfactin

Special structures of surfactin give it diverse biological functions that hold great potentials of being widely used in various fields. Potential applications and biological functions of surfactin have been briefly summarized in Table 1. We particularly focus on discussing potential applications and beneficial roles of surfactin in the fields of medical care and food safety in this section.

**Table 1.** Potential applications and biological functions of surfactin.

Potential applications	Biological functions	Refs.
Antibacterial activity	Suppress the expression of inflammatory mediators	[33]
	Biological control of Arabidopsis root infection	[51]
	Antibacterial activity against *Brachyspira hyodysenteriae* and *Clostridium perfringens*	[45]
Antiviral activity	Decreases the titer of herpes simplex virus (HSV-1)	[36]
	Activity on pseudorabies virus *in vitro*	[52]
	Inhibits invasion of epithelial cells by enveloped viruses	[53]
Antimycoplasma activity	Complete inactivation of mycoplasma in mammalian monolayer and suspension cell cultures.	[37]
Anticancer activity	Growth inhibition of MCF-7 human breast cancer cells	[40]
	Inhibits cancer progression through growth inhibition, cell cycle arrest, apoptosis and metastasis arrest	[41]
	Induced necrosis-like death of Huh 7.5 hepatocellular carcinoma cells	[54]
Microbial oil recovery	Effective in separating oil when used as an emulsifier	[18]
	Enhanced oil recovery by 9.2%	[55]
	Maintenance of physical and oxidative stability of O/W algal oil DHA emulsions	[56]
Food antistaling agent	Reduced the growth of *Salmonella enterica* in meat	[43]
	Helps yogurt maintain its sensory quality and extend its shelf life	[44]
	Reduces the growth of mesophilic bacteria and maintains the organoleptic state of the juice	[43]
	Extend the shelf lives of fruits, vegetables and grains	[45]
Maintenance of gastrointestinal homeostasis	Regulates the intestinal microbiota of broilers	[48]
	Improve the intestinal health of tilapia (*Oreochromis niloticus*)	[49]
	Elimination of *Staphylococcus aureus* colonizing the human intestine by inhibiting population sensing	[50]
Cosmetics field	Suitability of surfactin as powerful emulsifier in cosmetics	[57]
Bioremediation	Stimulating indigenous microorganisms for enhanced bioremediation of diesel contaminated soil	[58]
	Removal of heavy metals from contaminated soil and sediments	[59]

### 4.1. Antibacterial, antiviral and antimycoplasma activities

The action of surfactin with the bilayer confers its antibacterial effect. Recent studies have revealed that surfactin has an impact on how endotoxin (lipopolysaccharide, LPS) interacts with eukaryotic cells. This compound, which inhibits endotoxin activity, has the potential to become a new anti-inflammatory agent. Surfactin might lower plasma levels of endotoxin and nitric oxide in septic shock rats as well as suppress the expression of endotoxin-induced inflammatory mediators (IL-1 and iNOS) [33],[34].

The number of carbon atoms on the fatty acid chain of surfactin has a significant role in the inactivation of viruses, and it has been demonstrated that surfactin exhibits an antiviral impact, particularly against enveloped viruses [35]. Increased fatty acid hydrophobicity in surfactin typically demonstrates potent virus inactivation capabilities. Surfactin attaches to the lipid bilayer during inactivation, causing the viral proteins involved in viral adsorption and invasion to completely detach from the envelope and disintegrate. Surfactin was found to be more effective at inactivating enclosed viruses than unenveloped viruses in *in vitro* tests on the drug's effects on various viruses, particularly herpes simplex and retroviruses. In medium containing 5% fetal calf serum (FCS), surfactin was active at 25 mM, whereas surfactin at a concentration of 80 mM caused a decrease in herpes simplex virus (HSV-1) titer > 4.4 log10 CCID50/mL within 15 min [36].

Mycoplasma is the smallest free-living microorganism and is a specialized parasite that causes respiratory inflammation and diseases of the urogenital tract. In *in vitro* experiments, the proliferation rate and morphology of mycoplasma-contaminated mammalian cells were improved when surfactin was used [37]. In addition, the low toxicity of surfactin in *in vivo* models and its potential to prevent sexually transmitted diseases caused by genital tract mycoplasma infections are also of great importance.

### 4.2. Anticancer activities

Surfactin has been found to exhibit anticancer properties against several cancer cells, including the ability to limit cancer spread and have antiproliferative and apoptotic effects [38]. Surfactin-mediated programmed cell death, which is primarily produced by apoptosis, regulates tissue development and homeostasis *in vivo* and offers a possible method for treating cancer [39].

Breast cancer is a malignant tumor that poses a serious health risk to women, and the most extensive research has focused on surfactin's anticancer efficacy against breast cancer cells probably. It was shown that *B. subtilis* CSY 191-derived surfactin inhibited the growth of human breast cancer cells MCF-7 in a dose-dependent manner with an IC50 of 9.65 µM at 24 hours. Furthermore, compared to the surfactin produced by the CSY 191 strain alone, the high yield obtained by co-fermenting cheonggukjang (a Korean sauce) and CSY 191 strain further boosted the degree of anticancer activity by a factor of two [40].

In addition to breast cancer, surfactin has shown biological activities against colon, cervical and hepatocellular cancers. Recently, many studies have investigated the mechanism of its anticancer activity. It was shown that surfactin attenuated 12-O-tetradecanoylphorbol-13-acetate (TPA)-induced nuclear translocation and activation of nuclear factor-κB (NF-κB) and activator protein-1 (AP-1), thereby inhibiting cancer cell invasion and metastasis. In addition, surfactin significantly inhibited the expression and activation of matrix metalloproteinase-9 (MMP9), an enzyme that degrades almost all protein components in the ECM (extracellular matrix), disrupting the histological barrier to tumor cell invasion and playing a key role in tumor invasion and metastasis [38]. Moreover, the amphiphilic nature of surfactin makes them readily incorporated into nanopreparations, and the use of nanopreparations offers the advantage of optimized surfactin delivery for improved anticancer therapy [41].

### 4.3. Food antistaling agent

Due to its small molecular weight, thermal stability, water solubility, non-toxicity and lack of side effects, surfactin is not harmful to natural foods that require long term storage [42]. In a previous study, surfactin and iturin strongly decreased the growth of *Salmonella enterica* in meat by five orders of magnitude [43]. Studies have shown that antimicrobial peptides (especially surfactin) combined with preservatives can help yogurt maintain sensory quality and extend shelf life [44]. In addition, surfactin has been shown to have a bactericidal effect when added to milk. The lipopeptide produced by *B. subtilis* fmbJ greatly reduced the growth of mesophilic bacteria, slowed down rancidity and preserved the juice's outstanding organoleptic status in fresh watermelon [43]. Surfactin has also been shown in previous studies to extend the shelf lives of fruits, vegetables and cereals as a biological preservative with minimal toxicity and degradability [45]. These studies illustrate the powerful role of surfactin in food preservation and storage.

### 4.4. Maintenance of gastrointestinal homeostasis

As soil microbes are thought to be transient, unlike the popular probiotic *Lactobacillus*, representatives of the genus *Bacillus* were not thought to be a natural component of the human gut microbiota. Despite the fact that some of its members remain in the host for a very long time and create a wide variety of bioactive chemicals, most current investigations of the human intestinal microbiota typically do not take into account the special functional role of the transitory microbiota. Representatives of the genus *Bacillus* colonize the epithelium as food, water and probiotic preparations enter the gastrointestinal tract (GIT), obfuscating the distinction between the resident and transient microbiota. Although these bacteria represent a small proportion of the microbiome composition, they can have a significant impact on the gut microbiota and the whole body due to the large variety of secreted compounds they produce [46],[47].

In a recent study, the metabolite surfactin produced by *B. subtilis* was shown to be effective in improving growth performance, alleviating expression of intestinal inflammatory genes and having a regulatory effect on the intestinal microbiota in broilers [48]. In addition, the addition of antibacterial peptide surfactin to tilapia (*Oreochromis niloticus*) feed can improve intestinal health by increasing the height of intestinal folds, regulating intestinal flora and increasing intestinal antioxidant capacity [49]. A class of widely used lipopeptides (fengycins) produced by *Bacillus* has been shown to eliminate *Staphylococcus aureus* colonizing the human gut by inhibiting population sensing, a process by which bacteria respond to their population density by altering their genetic regulation [50]. These studies highlight the importance of probiotics derived lipopeptides in maintaining intestinal homeostasis and reducing infectious diseases.

The molecular mechanism behind probiotic efficacy has not yet been fully uncovered or is only partially understood. Future research aiming to decipher the mechanisms that determine the properties of bacterial probiotics will certainly expand the field of scientifically proven probiotics for medical use, which are considered promising alternatives for the prevention of gastrointestinal infections.

## 5. Biosynthesis of surfactin

### 5.1. Non-ribosomal peptide synthase

**Figure 1.**
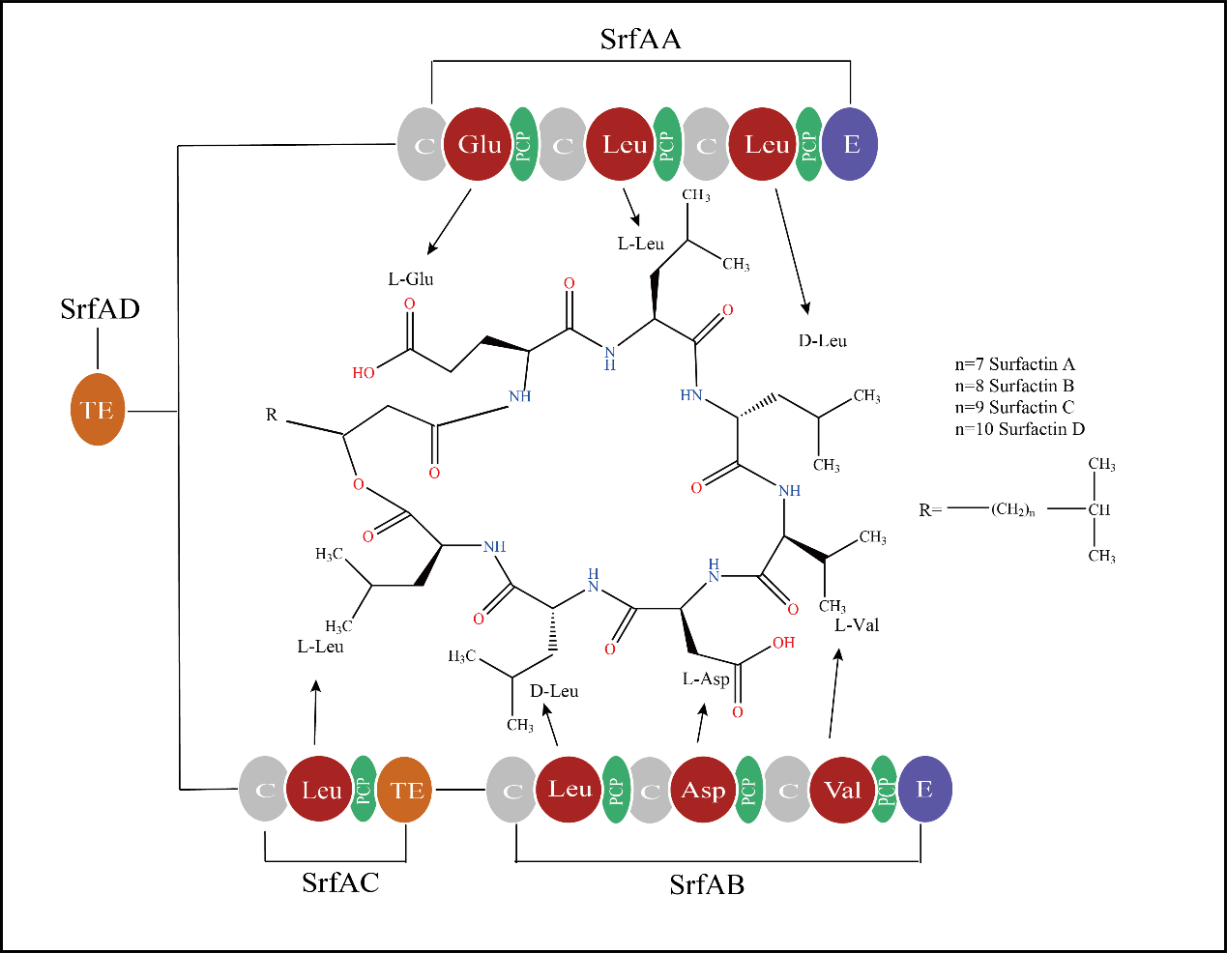
Non-ribosomal peptide synthetase modules for surfactin biosynthesis. *Note:* A, adenylation domain, represented by amino acids in red; PCP, peptidyl carrier protein domains, shown in green; C, condensation domain, shown in gray; E, epimerization domain, shown in blue; TE, thioesterase domain, shown in orange.

Surfactin is widely biosynthesized not only in *B. subtilis* but also in *Bacillus mojavensis*, *Bacillus licheniformis*, *Bacillus circulans*, *Bacillus nidulans*, and *Bacillus amylolyticus* [60]. Like most cyclic lipopeptides, surfactin is not synthesized by ribosomes but by a special system called non-ribosomal peptide synthase (NRPS) (Figure 1). SrfAA, SrfAB, SrfAC and SrfAD, which comprise a linear array of seven modules, are four modules that make up NRPS, and each is in charge of adding one amino acid [61],[62]. Each module has three catalytic structural domains at a minimum: an adenylation structural domain (A) that selects and activates substrates, a small peptidyl carrier protein (PCP) that transports aminoacyladenosine substrates as enzyme-bound thioesters and a condensation structural domain (C) that forms a peptide bond between acyl-S-PCP intermediates [63]. An extra thioesterase (TE) of the terminating module catalyzes the release of the product by hydrolysis or macrocyclization to produce cyclic or cyclic branched molecules after the epimeric (E) structural domain experiences a stereochemical conversion to produce a d-isomer of some bound residues [64].

### 5.2. Biosynthetic regulation

The synthesis of surfactin receives control from genetic regulation in addition to being influenced by the NRPS module. Surfactin synthesis is controlled by two genetic motifs, named *srf* and *sfp*. SRF is encoded by an inducible manipulator, the srfA motif (25 kb), and composed of four modular open reading frames (ORFs) [65], the function of which has been elucidated above. The second gene critical for surfactant production is *sfp*, located 4 kb downstream of the *srfA* manipulator [66]. *Sfp* encodes an enzyme belonging to the 4′-phosphopantetheinases family, which acts as a non-ribosomal peptide synthesis promoter. SFP enzymes can also convert the inactivated deoxygenated form of surfactin synthase into a holo-form containing cofactors, which can play an active role [67]. *SrfA* expression is induced at late exponential stages of bacterial growth and regulated by a number of transcriptional regulatory genes. *SrfA* expression is controlled by ComP-ComA in the quorum-sensing system, a mechanism that senses nutrient stress and regulates the expression of multiple genes [65].

The biosynthetic system of fatty acids, especially branched-chain fatty acids, also plays an important role in the synthesis of surfactin. The β-ketoacyl-acyl carrier protein synthase III (FabH) of *B. subtilis* has excellent activity and selectivity for branched-chain fatty acid synthesis precursors and can start the straight-chain and branched-chain fatty acid synthesis cycles by condensing acetyl-CoA, isobutyl-CoA, isopentyl-CoA, or -methylbutyl-CoA with malonyl ACP [68].

## 6. Strategies for enhancing microbial production of surfactin

Surfactin has been extensively studied for its antibacterial, antiviral, antitumor and hemolytic effects. However, due to its high economic cost and low synthetic yield, it cannot compete with chemically synthesized surfactants [6]. Although the current market for surfactin is estimated to grow at an annual rate of 2–4%, the high cost of production remains an issue limiting its large-scale synthesis. Moreover, its synthesis requires many steps and expensive mediator components, which also limits its development prospects [60]. Notably, despite certain advancements in the biosurfactant business, poor yields are still produced, and the downstream processes of recycling and purification have not yet achieved a sufficient economic scale [69]. In addition, the low rate of surfactin synthesis may be related to the complex regulation of biosynthesis, which results in production dependent on the cell density. This prevents continuous synthesis and limits the total yield [70].

## 7. Enhancement of surfactin biosynthesis by optimizing fermentation conditions

### 7.1. Fermentation process control

In recent years, research on improving surfactin yield by fermentation engineering has gradually increased. Fermentation parameters such pH, temperature, stirring rate, oxygen supply and medium composition are important factors to optimize, as they all affect the yield of surfactin. The surfactin yield of *B. subtilis* BS5 increased from 1.25 to 1.9 g/L when the pH was increased from 6 to 8 [71].

Temperature is also an important parameter of the culture medium. In the study of Amani et al., the yield of surfactin increased with increasing temperature, and the highest yield was at 37 °C [72]. The yield of surfactin varied when changing the agitation speed and oxygen supply of the medium, reaching a maximum yield of 6.45 g/L [25].

However, titers of most wild *Bacillus* strain-derived surfactin were below 5.0 g/L (Table 2). This is due to the antibiotic and signaling activity of surfactin itself, and this yield is not enough for large-scale production and industrial applications.

**Table 2.** Fermentation conditions for microbial production of surfactin.

Strains	Conditions	Modification	Surfactin production (g/L)	Refs.
*B. subtilis* BS5	pH	6.0	1.25	[71]
*B. subtilis* BS5		8.0	1.90	
*Bacillus* amyloliquefaciens		7.0	6.04	[73]
*B. subtilis* NLIM 0110	Temperature (°C)	25	1.75	[72]
*B. subtilis* NLIM 0110		30	3.20	
*B. subtilis* NLIM 0110		37	4.00	
*B. subtilis* NLIM 0110		45	3.50	
*B. subtilis* ATCC 21332	Agitation speed (rpm)	0	0.44	[25]
*B. subtilis* ATCC 21332		200	2.66	
*B. subtilis* ATCC 21332		250	4.44	
*B. subtilis* ATCC 21332		300	6.45	
*B. subtilis* ATCC 21332		350	1.01	
*B. subtilis* ATCC21332	Oxygen supply (vvm, L min^−1^)	1.50,3	6.45	[25]
*B. subtilis* ATCC21332		1.00,1	3.83	
*B. subtilis* ATCC21332		0.50,1	2.80	
*B. subtilis* BS5	Medium compositions	MSM plus FeSO_4_	0.40	[71]
*B. subtilis* BS5		MSM plus MnCl_2_	0.70	
*B. subtilis* BS5		MSM plus FeCl_3_	1.00	
*B. subtilis* BS5		MSM plus ZnSO_4_	1.75	

### 7.2. Developing new sources of carbon

In the biosurfactant industry, profitable product application is a major concern. Proper selection of substrates to be used in the production of biosurfactants is a fundamental prerequisite. Water-insoluble substrates are often used in biosurfactant production, due to the fact that microbial communities that produce biosurfactants are often isolated from petroleum and hydrocarbon-contaminated environments [69]. However, increasing evidence showed that water-soluble carbon sources, such as glucose, fructose and sucrose, can also be used as substrates for the synthesis of surface active substances from various microbial communities [74].

Despite the increasing number of reports on surfactin, the commercial production process remains somewhat difficult, mainly due to the increased cost of chemicals in the growth media used to produce surfactin. Cheap waste biomass is now being used as a substitute for refined carbon and nitrogen sources in order to reduce costs. For example, in a study of surfactin production using low-cost beer waste as a carbon source, *B. subtilis* produced surfactin at an amount of 210.11 mg/L for 28 hours, with inhibition activity against all tested bacteria, and complete inhibition was achieved against *Pseudomonas aeruginosa*, indicating that surfactin produced from low-cost substrates has the potential to be a promising bactericide [75]. In addition, a number of studies have been conducted on the production of surfactin from hydrolysate, glutamate, wine lees and other wastes with the aim of reducing its production cost by using cheap carbon sources, and the final yield of surfactin was around 500 mg/L [76],[77].

## 8. Enhancement of surfactin biosynthesis by genetic engineering

**Figure 2.**
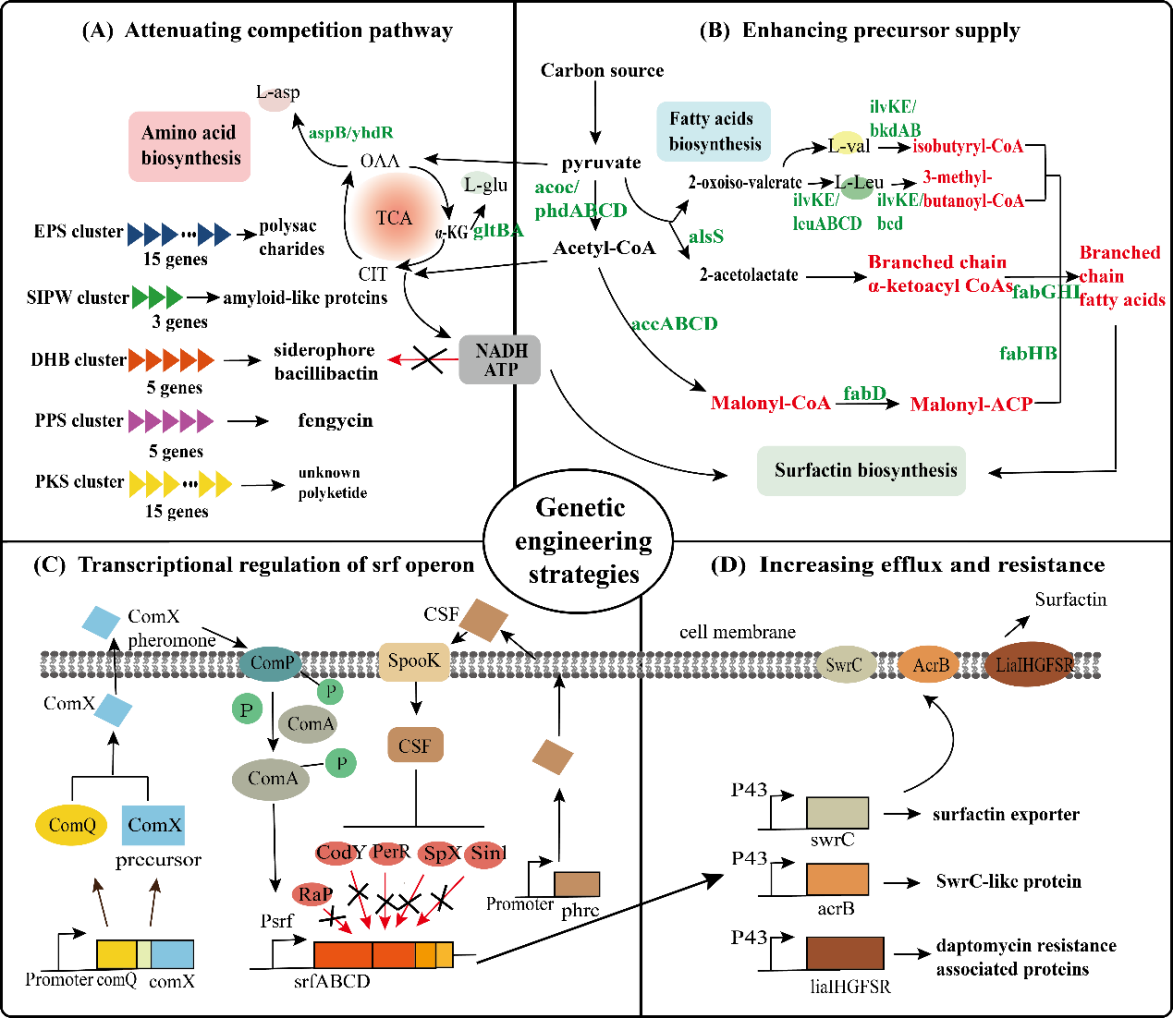
Genetic engineering strategies for enhancing microbial production of surfactin. *Note:* (A) Attenuating competition pathway module. The red arrow and the black cross indicate the deletion of pathways. (B) Enhancing precursor supply module. Overexpressed genes and important precursors are highlighted by green and red, respectively. (C) Transcriptional regulation of srf operon module. T-bar indicates the negative effects on DNA binding or protein interactions. “P” in the circle represents the phosphoryl group. A bent arrow represents the promoter. The red arrows and the black crosses indicate the deletion of negative transcriptional regulators. (D) Increasing efflux and resistance module. Specific roles of the proteins are listed following black arrows.

Based on a series of problems in surfactin production, the study aims to enhance the different stages of surfactin biosynthesis through advanced techniques to achieve higher surfactin yields by altering transcriptional regulatory genes and promoter substitutions, attenuating competition pathways, increasing precursor supply or enhancing secretion (Figure 2).

### 8.1. Enhancement of surfactin biosynthesis by promoter engineering

It has been well documented that surfactin production can be significantly increased using genetic engineering approaches (Table 3). Surfactin synthesis is critically regulated by the Psrf promoter, which controls the expression of the *srfA* operon. In the genetic modification of surfactin, promoter swapping has attracted much attention due to the challenge of heterologous expression of the *srfA* operon. As previously described, the four genes *srfAA*, *srfAB*, *srfAC* and *srfAD* are activated by signaling molecules of the quorum-sensing pathway under the control of the Psrf self-inducible promoter responsible for encoding the NRPS of surfactin [78].

In a previous study [85], four strong promoters, PgroE, Pcdd, PrplK and PsspE, were identified and cloned from the genome of *B. subtilis* THY-7. The optimal PgroE promoter was screened by single crossover homologous recombination, but the PgroE-containing strain was unable to synthesize surfactin. Subsequent replacement of PsrfA with the sucrose-inducible promoters PsacB and PsacP yielded engineered strains that produced 1.09 and 0.22 g/L of surfactin, respectively. By fusing the PgroE and PsacB ribonucleotide promoters (RAT), an artificial sucrose-inducible Pg1 promoter was generated, and the engineered strain containing the Pg1 substitution produced a surfactin titer of 1.44 g/L. An artificial IPTG-inducible promoter Pg2 was constructed from a PgroE-lacO fusion, which then replaced the PsrfA locus on the chromosome to yield THY-7/Pg2-srfA, whose surfactin titer increased to 5.98 g/L. On top of this, a new promoter Pg3 was generated by adding two point mutations in the -35 and -10 regions, which produced up to 9.74 g/L of surfactin, 15.6-fold higher than the original strain. In addition, a library of PsrfA derivatives was optimized by shortening the sequence of PsrfA and changing the nucleotides in the conserved regions of -35, -15 and -10 regions, using green fluorescent protein (GFP) as the reporter protein. The strongest promoter, P10, which was screened, had 150% higher GFP expression intensity than PsrfA and was able to efficiently heterologously express the exogenous protein [89]. As a tailored SrfA expression promoter for each strain may be more appropriate, it is crucial to investigate efficient promoters to increase surfactant production.

**Table 3.** Genetic manipulations for improving surfactin yield.

Strains	Modification	Surfactin production (g/l)	Refs.
Branched chain fatty acids biosynthesis			
*B. subtilis* 168S23	Promoter substitution of key genes for precursor material	8.5	[79]
*B. subtilis* TS1726	Overexpression of key genes	13.37	[80]
Branched chain amino acids biosynthesis			
*B. subtilis* H1	Inhibition of negative transcriptional regulators	0.75	[81]
*B. amyloliquefaciens* MT45	Upregulation of synthetic genes in L-glu / L-asp	2.93	[70]
*B. subtilis* TS1726ΔspoIVB	Overexpression of key genes and medium optimization	16.7	[82]
Surfactin efflux			
*B. subtilis* 168S12	Overexpression of transporter proteins	3.8	[83]
*B. subtilis* TS662	Overexpression of transporter genes	1.67	[84]
Promoter engineering			
*B. subtilis* THY-7/Pg3-srfA	Replacement of PsrfA with Pg3	9.74	[85]
*B. subtilis* fmbR-1	Replacement of PsrfA with Pspac	3.86	[86]
Regulation of transcription factors			
*B. subtilis* TS1726ΔspoIVB	Deleting regulator of sporulation	9.6	[82]
*B. subtilis* (pHT43-comXphrC)	Overexpression of positive regulators	0.135	[87]
*B. subtilis* 168S35	Overexpression of positive regulators and deletion of negative regulators	12.8	[83]
Genome reduction			
*B. amyloliquefaciens* GR167	4.18% reduction of genome	9.7% increased	[88]

### 8.2. Regulation of transcriptional factors

The expression of the *srfA* operon is controlled by a number of transcriptional regulatory genes in addition to the promoter. ComX and CSF are two typical peptides that control quorum-sensing in *B. subtilis*. By phosphorylating the transcription factor ComA, ComX and CSF each use a different mechanism to start the *srfA* operon's transcription (ComA-P) [90],[91]. In previous studies, the expression of *srfA* was successfully reduced by repressing the quorum-sensing system ComQXP transcription in *B. subtilis*. Overexpression of the two signal factors encoded by *comX* and *phrC* in *B. subtilis* could stimulate transcription of the *srfA* operon, thereby increasing the production of surfactin [87],[92].

Multiple global regulators and proteins also control the expression of the *srfA* operon. CodY can restrict *srfA* transcription by binding directly to the *srfA* promoter area, which can be activated by high amino acid concentrations. Inactivation of *codY* could increase surfactin production in *B. subtilis* 168 by roughly 10-fold [93]. In addition, other negative regulators of *srfA* have been studied in detail and reported, such as *sinI*, *spX, raP* and *perR* [70],[83],[94]. Moreover, the synthesis of surfactin was increased after knocking out spore synthesis-related genes [82]. This may be related to the utilization of nutrients by spore synthesis. At present, quorum-sensing directed regulatory network needs to be further investigated, and more signal peptides and transcriptional regulators of surfactin synthesis need to be explored.

### 8.3. Increasing precursor supply for surfactin synthesis

Different metabolic engineering techniques, such as (i) increased supply of branched-ketoacyl-CoA, (ii) increased synthesis of malonyl-ACP and (iii) overexpression of the whole fatty acid synthase complex, were used to augment surfactin production in terms of branched fatty acid supply.

The catalytic interaction of branched-chain α-keto-CoA with malonyl-ACP by FabHB (β-ketoacyl-carrier protein synthase III) initiates the synthesis of branched-chain fatty acids. In order to increase the intracellular content of branched α-keto CoA to increase the supply of branched fatty acid biosynthetic precursors, the branched α-keto acid dehydrogenase complex (BKD) is overexpressed. BKD is a complex manipulator of branched keto acid biosynthesis, because the dehydrogenase activity of BKD requires lipidation. Overexpression of BKD in *B. subtilis* may compete with other lipoic acid-dependent complexes for lipidation of proteins and inhibits cell proliferation and surfactin production. It has been shown that lipoic acid biosynthesis in *B. subtilis* is dependent on three enzymes, LIPA, LIPL and LIPM. Overexpression of lipALM to eliminate the competitive lipoylation process between BKD and other lipoic acid-dependent complexes led to a surfactin yield of 4.6 g/L, an increase of 21% compared to the parental strain [83]. Another targeting pathway for modification is the synthesis of malonyl-ACP. In the fatty acid biosynthetic pathway, the acetyl CoA carboxylase complex (ACCD, ACCA, ACCB, ACCC) catalyzes the conversion of acetyl-coA to malonyl-coA. FabD (malonyl-coA: acyl carrier protein transacylase) then converts the malonyl-coA to malonyl-ACP, starting the synthesis and expansion of the fatty acid chain. According to these findings, overexpression of the endogenous *accDABC* and *fabD* increased surfactin production by 14% [83]. FBF (β-ketoacyl-acyl carrier protein synthase II), FBG (β-ketoacyl-acyl carrier protein reductase), FBZ (β-hydroxyacyl-acyl carrier protein dehydratase) and FBI made up the fatty acid synthase complex (cyclic enoyl-acyl carrier protein reductase). After overexpressing the fatty acid synthase complex with increasing supplies of both branched-ketoacyl-coA and malonyl-coA, the surfactin production was further raised to 8.5 g/L. However, if these interventions are combined with transcriptional enhancement of the *srf* gene cluster, surfactin production can be further increased to 12.8 g/L, reaching 42% of the theoretical production [83].

In addition to fatty acids, amino acids are also important precursors for the biosynthesis of surfactin. The genes *yrpC*, *racE* or *murC* for L-glutamate synthesis and *bkdAA* and *bkdAB* for L-leucine and L-valine synthesis were inhibited by CRISPRi technology, reducing the metabolic flux of competing amino biosynthetic pathways, which increased the production of surfactin and the proportion of C14 subtypes [81]. After overexpression of the gene for the leucine pathway in the non-spore producing strain TS1726, more leucine (5 g/L) was added, and the surfactin titer reached 16.7 g/L [82].

### 8.4. Enhancement of surfactin biosynthesis by attenuating competition pathways

In *B. subtilis* 168, although biofilm synthesis is not possible, the gene clusters *epsA-O* and *tasA-sipW-yqxM*, which are associated with biofilm synthesis, still have high transcriptional activity. *EpsA-O* and *tasA-sipW-yqxM* are responsible for polysaccharide and amyloid synthesis, respectively, in *B. subtilis*. Xu et al. knocked out the *eps* gene operon and the *tasA-sipW-yqxM* operon, respectively, and surfactin production increased 1.8 and 1.3-fold, respectively. When both operons were knocked out simultaneously, the surfactin yield reached 1.4 g/L, which was 2.5-fold higher than the original. On top of this, further knockdown of the gene clusters *pps*, *dhb* and *pks*, encoding nonribosomal peptide fengycin, the siderophore bacillibactin and an unknown polyketide, increased the surfactin yield by about 3.3-fold [83]. This is probably because the biosynthesis of these different proteins, lipopeptides and polyketides will inevitably compete with surfactin production for energy, NADH and direct precursors.

With the development of systems biology, homologous recombination and genome reduction techniques have become popular research topics for the construction of various chassis cells. In a previous study, genome reduction in *B. amyloliquefaciens* LL3 strain deleted about 4.18% of non-essential genes, reducing the competition for surfactin precursor material and energy, leading to a significant increase in surfactin production [88].

### 8.5. Enhancing the expression of surfactin export proteins

Tsuge et al. determined the correlation between the expression level of the *yerP* gene and the resistance of *B. subtilis* to surfactin, and they hypothesized that *yerP* was associated with surfactin efflux. Although there was no direct evidence that YerP can cause surfactin efflux, the inherent metabolite surfactin of *B. subtilis* severely inhibited the growth of YerP-deficient strains, suggesting that the enhanced susceptibility of mutant strains to surfactin was closely linked to the dysfunction of YerP [95]. Li et al. showed that overexpression of three lipopeptide transporters (YcxA, KrsE and YerP), the functioning of which depended on proton motive force, resulted in 89%, 52% and 145% increases in surfactin export, respectively [84]. Although the mechanism of self-resistance in surfactin-producing strains was not well known, the production of surfactin was increased to 3.8 g/L after overexpression of resistance proteins SwrC, AcrB and LiaIHGFSR [83]. These studies hold promise for a better understanding of surfactin efflux.

## 9. Enhancement of surfactin biosynthesis by synthetic biology directed rational design

With the development of synthetic biology, various genes have been heterologously expressed, leading to successfully heterologous biosynthesis of various molecular compounds [96]. Studies on heterologous expression of surfactin in non-*Bacillus* hosts have not been reported, as cloning of large DNA fragments and stable expression of heterologous biosynthetic genes remain challenging [97]. Several synthetic biology tools have been developed to clone entire biosynthetic gene clusters directly from complex genomes, such as tools for yeast transformation-associated recombination (TARs) [98], yTREX systems [97] and multiplex CRISPR-TAR [99] for promoter engineering. These tools have been widely used to clone and modify biosynthetic genes, including some genes related to surfactin synthesis. In the future, synthetic biology tools could be used to engineer more stable genes to ensure efficient expression of biosynthetic gene clusters and to replace traditional surfactin-producing hosts.

In the future, a new research direction is to use synthetic biotechnology and metabolic engineering to improve the ability of strains to obtain surfactin from xylose as the sole carbon source. When xylose was used as the sole carbon source, *B. subtilis* 168 reduced organic acid by-products and was able to efficiently produce surfactin (up to 2.074 g/L). However, the utilization of xylose is limited by the production characteristics of the strain and enzyme and the inhibition of AraE by the AraR protein [100]. Hu et al. found that *B. subtilis* 168 could effectively use xylose to eliminate the inhibition of AraE by AraR when combined with organic nitrogen sources. Recombinant strain BSFX022 increased the supply of precursor fatty acyl CoA by overexpressing *sfp* and *bte* genes and *yhfl* gene, thereby overexpressing 4′-phosphopanthenol transferase, medium-chain acyl-acyl carrier protein (ACP) thioesterase and fatty acyl CoA ligase, ultimately producing 2.203 g/L of surfactin [101].

**Figure 3.**
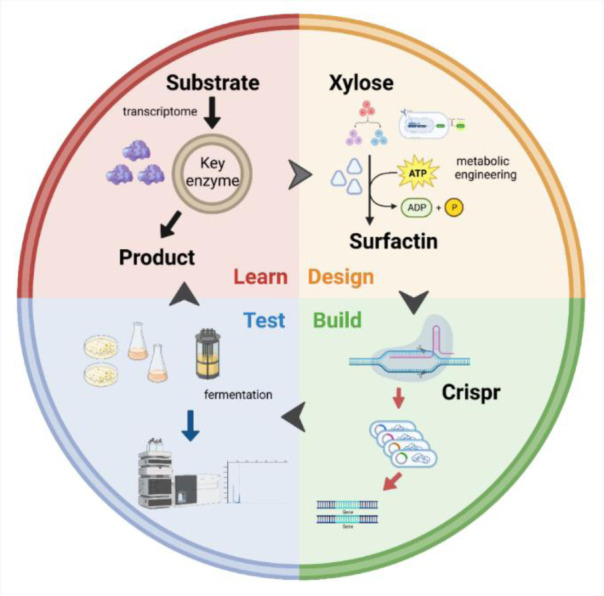
Synthetic biology and metabolic engineering of xylose utilization as exemplified by Crispr technology.

Nevertheless, *B. subtilis* using xylose as the sole carbon source for surfactin synthesis displayed some shortcomings, such as the complex and rigorous mechanism of CCR (carbon catabolite repression) and insufficient metabolic regulation of xylose utilization. A new research direction is to use synthetic biotechnology combined with metabolic engineering to gradually improve xylose metabolism in the host and enhance the ability of the strain to produce surfactin. An “optimization” approach for this pathway was established by applying synthetic biology design principles (learn, design, build and test) (Figure 3) and transcriptome optimization of the target bacterial gene cluster, combined with optimization of promoters, RBS (ribosome binding sites), terminators and other components in the context of metabolic engineering, promising further improvements in surfactin production [102].

*Caulobacter crescentus* has been found to be able to metabolize xylose efficiently, and many studies have been conducted to establish a new pathway for xylose utilization by heterologously expressing its related enzymes in other microorganisms [103]. Related approaches include evolutionary engineering, which identifies mutations that favor the growth of *B. subtilis* on xylose. These mutations would be designed to derepress AraR and produce recombinant *B. subtilis* that could utilize xylose for rapid growth. The strains were designed to effectively improve xylose conversion efficiency. Screening for xylose-specific transporter proteins by enhancing the expression of the transporter protein AraE and designing homologous or heterologous transporter proteins can also greatly improve xylose utilization. In conclusion, the continued development of synthetic biology will advance research on the production of surfactin from xylose, thus providing another option for inexpensive industrial production.

## 10. Conclusions and prospects

Surfactin has stimulated a lot of interest because of its specific structure and biological activity, including its activity in microbial oil recovery and emulsification properties. This paper reviews the potential applications and beneficial effects of surfactin, such as antibacterial effects, antiviral effects, anticancer effects, application in food preservation and maintenance of gastrointestinal homeostasis. However, although surfactin has great medical and commercial value, achieving its industrial production has been a challenge because of the high cost and low yield of its synthesis. At present, the highest titers of surfactin by microbial production could reach up to 16.7 g/L according to the most updated report [82], which offers a robust strategy combining genetic engineering and fermentation optimization for re-modeling *B. subtilis* to further improve its fermentation efficiency and industrial application for surfactin production. Here, we describe the non-ribosomal peptide synthesis mode of surfactin synthesis and the transcriptional regulators that regulate its synthesis. We also highlight some of the latest strategies that could be used to improve the yield of surfactin, including fermentation engineering approaches, rational genetic engineering and the combination of synthetic biology and metabolic engineering.

Notably, the establishment of suitable hosts for exogenous expression of surfactin biosynthetic genes to provide chassis strains for achieving efficient surfactin production should be an important direction for future research. Meanwhile, with the development of systematic biology, modeling of metabolic networks will help us to explore the interactions between metabolites and key metabolic modules and identify more information that may be useful for surfactin production. In addition, it has been found that the abundance of surfactin in the medium decreases as fermentation proceeds to the late stages of incubation (nutrient depletion), which may be related to an increase in protease activity [104]. The degradation process of surfactin has been little studied, and understanding its mechanism might also be important to further enhance microbial biosynthesis of surfactin for industrial production.
